# On the caveats of a multiplex test for SARS-CoV-2 to detect seroconversion after infection or vaccination

**DOI:** 10.1038/s41598-022-14294-8

**Published:** 2022-06-20

**Authors:** Lorena O. Fernandes-Siqueira, Fabiana A. P. Ferreira, Bruna G. Sousa, Nathane C. Mebus-Antunes, Thais C. Neves-Martins, Fabio C. L. Almeida, Gustavo C. Ferreira, Didier Salmon, Luciana S. Wermelinger, Andrea T. Da Poian

**Affiliations:** 1 Institute of Medical Biochemistry Leopoldo de Meis, Federal University of Rio de Janeiro, Rio de Janeiro, RJ 21941-902 Brazil; 2 National Center for Structural Biology and Bioimaging (CENABIO), Federal University of Rio de Janeiro, Rio de Janeiro, RJ 21941-590 Brazil; 3 School of Pharmacy, Federal University of Rio de Janeiro, Rio de Janeiro, RJ 21941-902 Brazil

**Keywords:** Immunological techniques, Viral infection

## Abstract

The Covid-19 pandemic, caused by SARS-CoV-2, has resulted in over 6 million reported deaths worldwide being one of the biggest challenges the world faces today. Here we present optimizations of all steps of an enzyme-linked immunosorbent assay (ELISA)-based test to detect IgG, IgA and IgM against the trimeric spike (S) protein, receptor binding domain (RBD), and N terminal domain of the nucleocapsid (N-NTD) protein of SARS-CoV-2. We discuss how to determine specific thresholds for antibody positivity and its limitations according to the antigen used. We applied the assay to a cohort of 126 individuals from Rio de Janeiro, Brazil, consisting of 23 PCR-positive individuals and 103 individuals without a confirmed diagnosis for SARS-CoV-2 infection. To illustrate the differences in serological responses to vaccinal immunization, we applied the test in 18 individuals from our cohort before and after receiving ChAdOx-1 nCoV-19 or CoronaVac vaccines. Taken together, our results show that the test can be customized at different stages depending on its application, enabling the user to analyze different cohorts, saving time, reagents, or samples. It is also a valuable tool for elucidating the immunological consequences of new viral strains and monitoring vaccination coverage and duration of response to different immunization regimens.

## Introduction

Severe acute respiratory syndrome coronavirus 2 (SARS-CoV-2) was first identified in December 2019 in Wuhan, China, and has rapidly spread worldwide, as the causative agent for the current Covid-19 pandemics (COronaVIrus Disease 2019)^1,2^. This betacoronavirus is an enveloped positive-sense single-stranded RNA virus formed by four structural proteins: nucleocapsid (N), membrane (M), envelope (E), and spike (S). SARS-CoV-2 entry into the target cells involves the binding of a region of S protein, the receptor binding domain (RBD), to the cell surface angiotensin-converting enzyme 2 (ACE2)^3^. Most infected people have cold-like symptoms or are asymptomatic. However, in some individuals, infection may result in a multisystem disease, progressing mainly to an acute respiratory distress syndrome (ARDS). So far, over 6 million deaths have been associated to Covid-19 around the world^4^.

Social distancing, use of masks and immunization are the most effective strategies to control the dissemination of the disease^5–7^. Therefore, populational testing is critical to accurately monitor the course of the disease and drive policies for the mitigation of the disease outcomes^8^. Additionally, there is a latent risk of the emergence of new variants^9^ with greater virulence or for which the effectiveness of the vaccines is reduced. These factors pinpoint the importance of customizable tests for the antigens of interest, allowing a quick and effective response in monitoring infection, as well as contributing to the elucidation of the immunological consequences of the new strains.

Serological tests vary in their individual performance characteristics. Currently, they are not recommended to assess for immunity to SARS-CoV-2 following vaccination since some of the tests do not detect the antibodies generated by Covid-19 vaccines^10^. In this context, a wide variety of assays to detect immunological reactivity against SARS-CoV-2 antigens are still being developed, tested, and applied. It is important to highlight the high costs of the commercial SARS-CoV-2 serological kits, which also can limit their use^11^.

Here, we describe an efficient in-house enzyme-linked immunosorbent assay (ELISA) that assesses the presence of three different antibody isotypes, IgA, IgM and IgG, directed against multiple SARS-CoV-2 antigens used in the diagnosis of Covid-19 (the trimeric spike protein, S; the S protein receptor binding domain, RBD; and the N terminal domain of the nucleocapsid protein, N-NTD). Some possible applications of the developed assay are also presented.

## Results

### Choice of antigens

Most serological diagnostic tests for SARS-CoV-2 use S and N proteins as the main antigens due to their high immunogenic characteristic^11,12^. In addition, serum reactivity to RBD has also been reported as a good predictor of SARS-CoV-2 infection^13–15^. For the assay described here, we used three different antigens: the trimeric S; the RBD (residues 319 to 541 of S protein); and the N-terminal domain of N protein (residues 44 to 180 of N protein; N-NTD) (Fig. S1). We evaluated separately the reactivity of 3 immunoglobulins (IgG, IgA and IgM) to each of the antigens (S, RBD, N-NTD), so that the test provides 9 different reactivity results for each serum.

### Assay development

**Figure 1:**
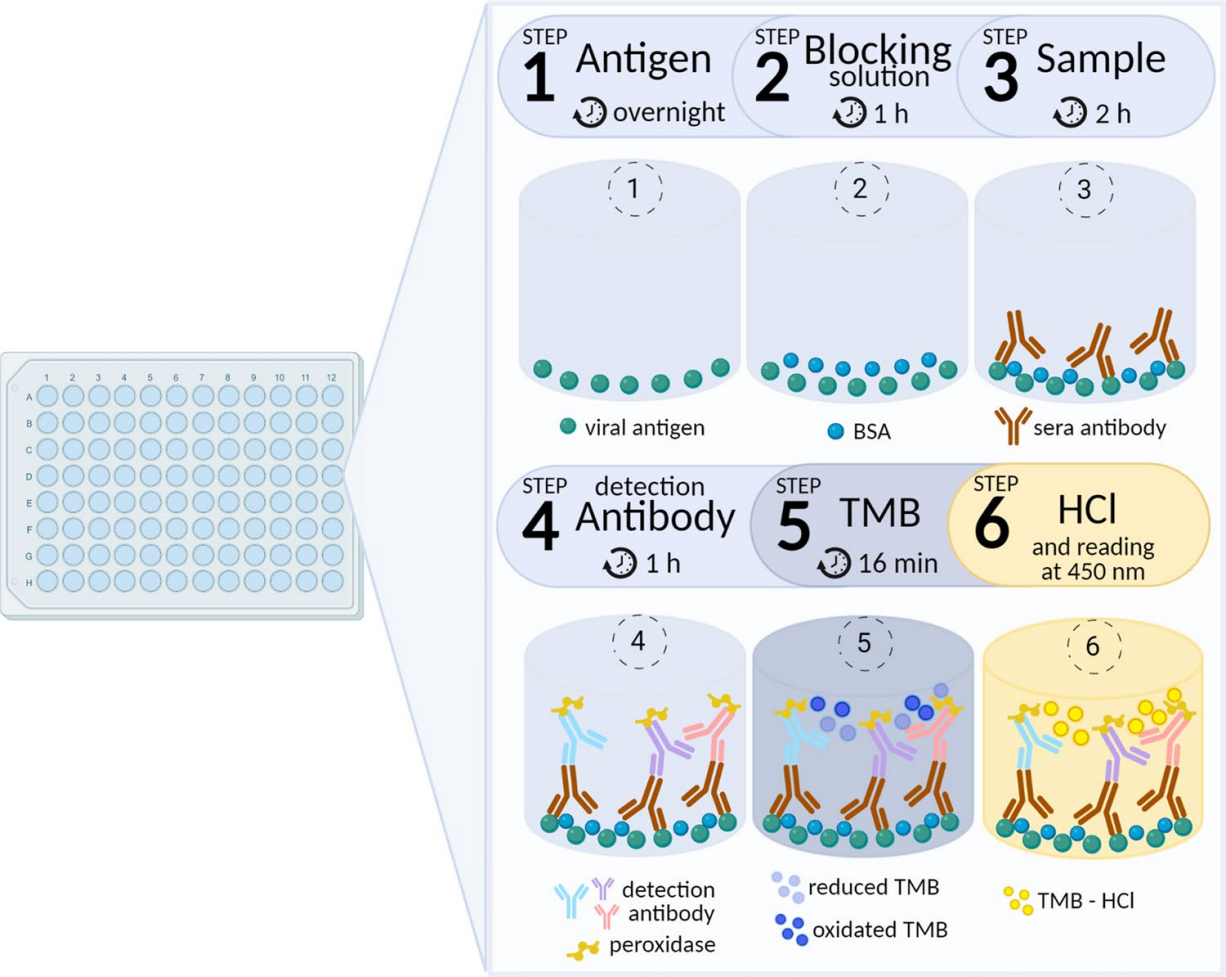
Scheme summarizing the assay steps. Step 1 corresponds to an overnight plate coating with each of the three SARS-CoV-2 antigens used in the assay—S, RBD and N-NTD recombinant proteins. In the step 2, the empty spaces on well surface are filled with 3% BSA solution (blocking solution). In the step 3, serum samples are added to allow the specific binding of sera immunoglobulin to the antigen. Step 4 corresponds to the addition of peroxidase-conjugated antibodies that specifically bind to sera IgG, IgA or IgM bound to the plate. Step 5 correspond to the addition of the chromogenic reagent TMB that is reduced as peroxidase reaction proceeds, developing a blue color. In step 6 peroxidase reaction is stopped by HCl addition, which also converts blue TMB in a yellow compound that is quantified spectrophotometrically at 450 nm. Created with BioRender.com.

The assay presented here was adapted from a previously reported protocol^16^ to allow the analysis of 3 types of serum antibodies—IgG, IgA, and IgM—against 3 SARS-CoV-2 antigens—trimeric Spike (S), RBD and N-NTD. To set the best assay conditions, we varied the antigen coating density, sera and detection antibodies’ concentration, as well as the incubation time with blocking solution, sera, detection antibodies and chromogenic substrate for assay development. A schematic representation of the complete assay is shown in Fig. 1.

To determine the best conditions of plate coating with the viral antigens, and the appropriate dilutions of sera and secondary antibodies, we used serum samples from 23 individuals with positive PCR result for SARS-CoV-2, as well as three sera collected before SARS-CoV-2 pandemic (Table 1). Since PCR + samples reacted differently with each of the antigens, the 4 most reactive samples for a given antigen were chosen for the optimization of the respective test.

**Table 1:** Cohort characteristics.

Characteristics		Pre-pandemic (n = 42)	Asymptomatic (n = 63)	Symptomatic without PCR confirmation (n = 40)	PCR-positive (n = 23)	Vaccinated (previously non-reactive) (n = 11)	Vaccinated (previously reactive to S IgG) (n = 7)
Seroconversion (IgG), %	Spike (S)	ND	8	17	100	100	100
	RBD	ND	8	11	78	100	100
	N-NTD	ND	14	17	52	0	60
Age, average (range)		38 (19—70)	33 (20—66)	36 (24—61)	37 (21–62)	43 (27–59)	27 (22–36)
Sex, %	Female	43	66	68	75	73	86
	Male	57	34	32	25	27	14
Main symptoms, %	Fever	NA	ND	40	25	NA	0
	Cough	NA	ND	55	46	NA	14
	Anosmia/ageusia	NA	ND	27	46	NA	57
	Sore throat	NA	ND	60	37	NA	14
	Sneezes	NA	ND	62	37	NA	28
	Nasal congestion/runny nose	NA	ND	67	37	NA	71
	Headache	NA	ND	45	46	NA	28
	Chills/myalgia	NA	ND	22	33	NA	57
	Gastrointestinal symptoms	NA	ND	25	33	NA	28
Days post PCR at collection date, average (range)		NA	NA	NA	51 (10—167)	NA	NA
Vaccine, %	CoronaVac	NA	NA	NA	NA	36	29
	ChAdOx1 nCoV-19	NA	NA	NA	NA	64	71

*ND* not detected;* NA* not applicable.

#### Antigen coating density

**Figure 2:**
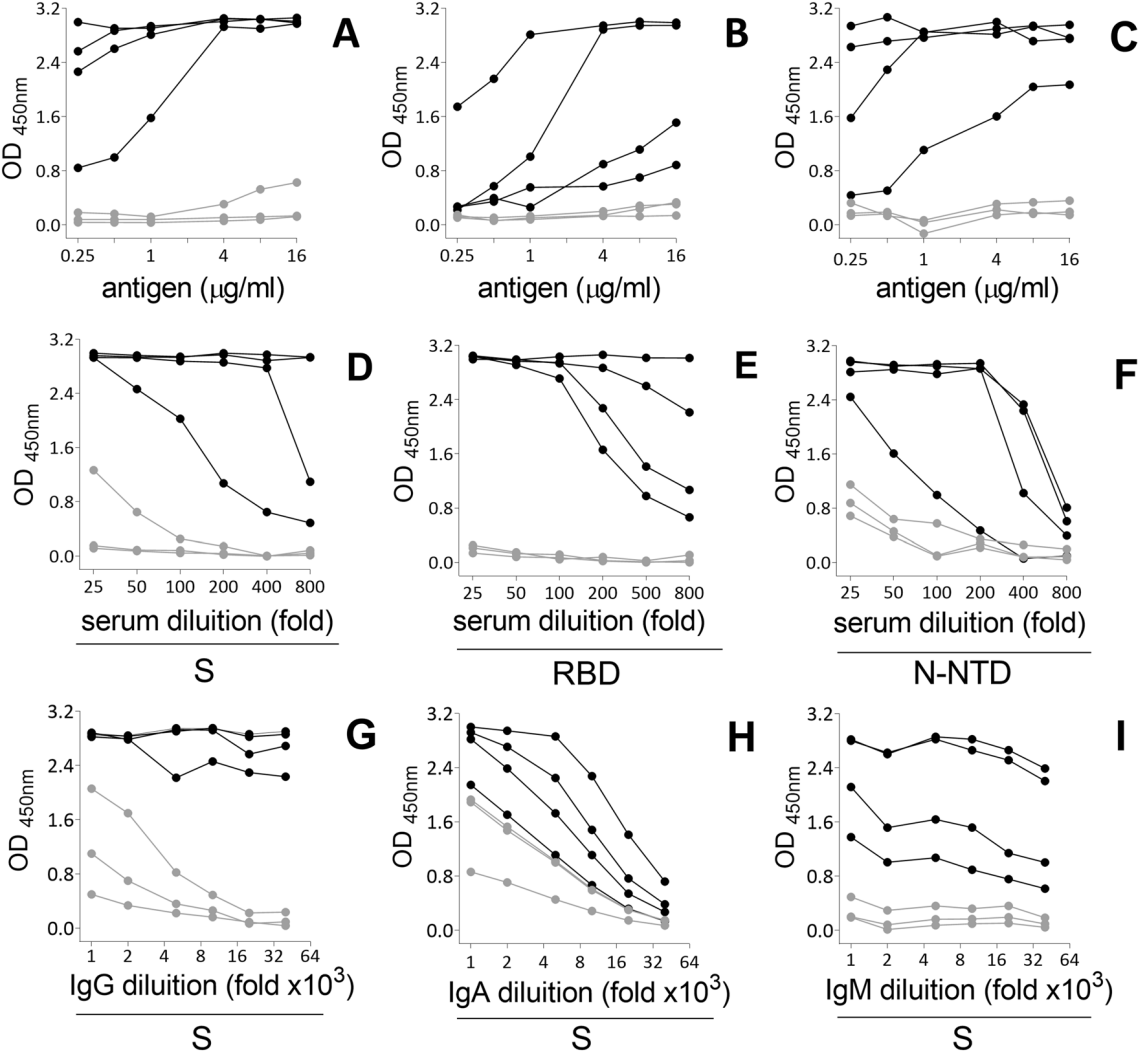
Sera reactivity to SARS-CoV-2 antigens in different assay conditions. (**A**–**C**) Effect of antigen density: SARS-CoV-2 antigens S (A), RBD (B), or N-NTD (C) were serially diluted in PBS and used as antigens to coat ELISA plates in assays in which sera were used at a 1:50 dilution. (**D**–**F**) Effect of sera concentration: sera samples were serially diluted in a 1% BSA solution in PBS-T and assay was conducted using plates previously coated with 4 µg/ml of S (D), RBD (E) or N-NTD (F). (**G**–**I**) Effect of secondary antibody dilution: reactivity of serum IgG (G), IgA (H) or IgM (I) to S protein was accessed using the respective detection antibody serially diluted in a 1% BSA solution in PBS-T, in a plate previously coated with 4 µg/ml S, incubated with the sera samples at a 1:50 dilution. For all the conditions tested, the plates were incubated with the antigens overnight, at 4 °C, blocked for 1 h with 3% BSA in PBS-T and incubated for 2 h with 3 sera collected before pandemic (grey symbols) or 4 sera from PCR + individuals (black symbols). Sera reactivity was quantified spectrophotometrically after incubation with the respective detection antibody for 1 h followed by the addition of the chromogenic substrate.

For an in-house ELISA, determining the ideal antigen concentration to coat the plates is particularly important when the antigens are also produced in-house. To define the best antigen coating density, we performed two-fold serial dilution of each antigen (S, RBD and N-NTD), ranging from 0.25 to 16 µg/ml, with 50 µl of antigen solution applied per well (12.5 to 800 ng antigen per well). For each antigen, tests were conducted using the 4 most appropriate PCR + and 3 pre-pandemic samples assayed for either IgG (Fig. 2A–C), IgA or IgM detection (Fig. S2). We observed no reactivity of pre-pandemic sera at any condition tested, while a dose-dependent antigen recognition profile was identified for most PCR + samples. We then chose the concentration of 4 μg/ml for all antigens to perform the subsequent assays.

To evaluate the actual contribution of RBD to sera reactivity to S, we performed an assay coating the plates with S or RBD with the concentrations adjusted to maintain the same amount of RBD epitopes. Since S and RBD molecular masses are approximately 180 kDa and 26 kDa, respectively, we compared sera reactivity using 4 μg/ml S with 0.6 μg/ml RBD (RBD molar equivalent in the complete S) or 4 μg/ml RBD (maintaining the same protein mass) (Fig. S3). We observed a high reactivity to RBD even when used at a lower concentration. This result may be associated with a large amount of sample antibodies directed to the RBD, suggesting that this domain has a major contribution in the reactivity against the entire S. No significant changes were observed in the results of PCR + samples for IgG when 4 or 0.6 μg/ml RBD concentration were used, suggesting that using 0.6 μg/ml RBD is enough to ensure reliable results for this immunoglobulin. However, for the IgA and IgM detection assay, a better separation of positive samples was revealed when 4 μg/ml RBD was used.

#### Sera dilution

To determine the best sera concentration to be used in the assay, we performed two-fold serial dilution of the 4 most appropriate PCR + and 3 pre-pandemic sera samples, with dilutions ranging from 1:25 to 1:800. Assays were performed by using 4 μg/ml of the antigens to coat the plates, assessing reactivity to either IgG (Fig. 2D–F), IgA or IgM (S4). We found that, especially for IgG reactivity against all the antigens (Fig. 2D–F) and for all antibodies tested against N-NTD (Figs. 2F and S4E,F), dilutions lower than 1:50 result in a substantial increase in the background of some negative samples, which may generate false positive results. For most cases of PCR + samples, the ideal dilution was between 1:50 and 1:100 (to reach values that allow a good separation of positives from negatives samples).

**Figure 3:**
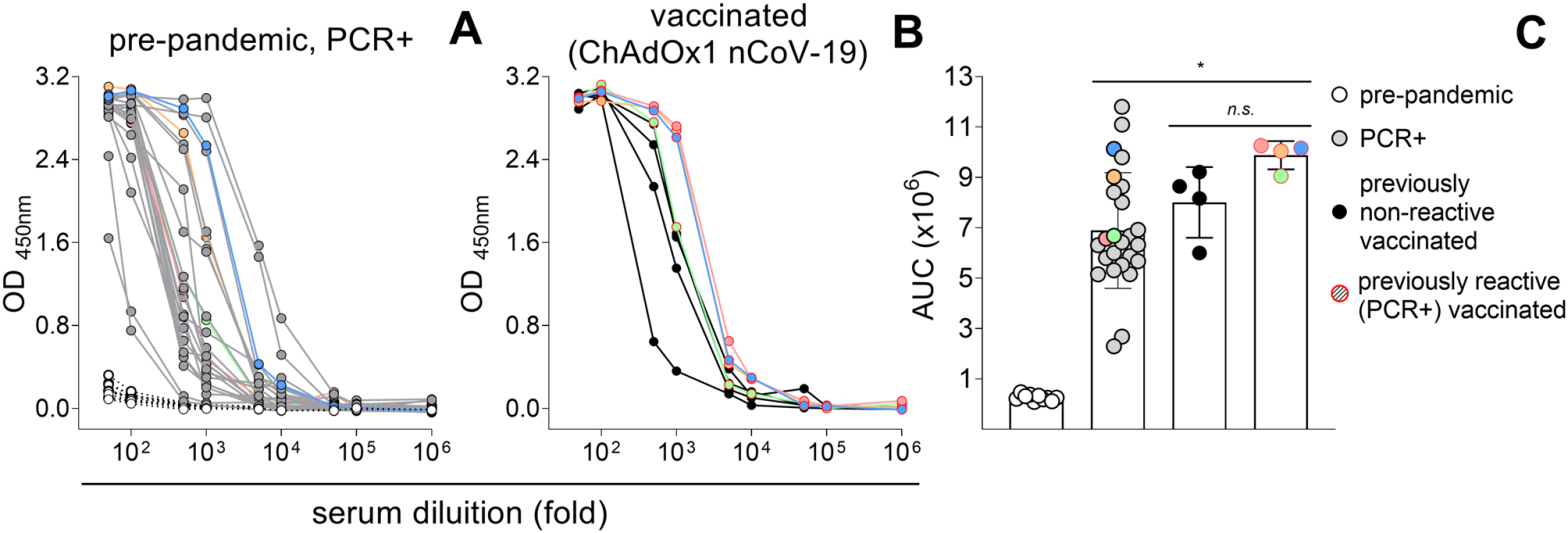
Titration of serum antibodies against SARS-CoV-2 S protein. Sera from 10 pre-pandemic (white symbols) and 23 PCR + (grey symbols) individuals (**A**) or sera from individuals vaccinated with two doses of ChAdOx1 nCoV-19 who were reactive (PCR +) (colored symbols) or not (black symbols) before vaccination (**B**) were serially diluted in a 1% BSA solution in PBS-T and incubated for 2 h in plates previously coated with 50 μl solution of S protein at 4 μg/ml, overnight, at 4 °C, and blocked for 1 h with 3% BSA in PBS-T. IgG reactivity was quantified spectrophotometrically after incubation with the respective detection antibody for 1 h followed by the addition of the chromogenic substrate. The values for the area under the curves (AUC) obtained for each sample are represented in (**C**). * *p* = 0.004, *n.s*. = non significative.

To evaluate quantitatively the assay sensitivity, we broadened the sera dilution range (tenfold serial dilutions) using three different groups of positive samples: (i) 23 PCR + sera collected before vaccination has started (PCR +); (ii) 4 sera from individuals vaccinated with two doses of ChAdOx1 nCoV-19 vaccine who were not reactive before vaccination (Vaccinated—previously non-reactive); and (iii) 4 sera from individuals vaccinated with two doses of ChAdOx1 nCoV-19 vaccine who were reactive before vaccination (Vaccinated—previously S IgG reactive). As negative controls, we used 10 sera samples collected before pandemic. Sera titration revealed that reactive samples may show very different antibody titers (Fig. 3). Calculations of the area under the curves (AUC) showed that sera from vaccinated individuals who tested positive before vaccination presented significantly higher titers when compared to sera from vaccinated individuals who were non-reactive when receiving the first vaccine dose. It is also clear the high antibody titer variability among the pre-vaccination PCR + sera (Fig. 3A,C). This variability would be explained by the timing, severity of symptoms and longevity of the humoral response against SARS-CoV-2, as those sera were collected in different time points after individuals were infected, or by differences in the response intensity within a population. Additionally, individuals with lower titers before vaccination had significantly greater increases in antibody titers when compared to individuals with high titers before vaccination (Fig. 3C, colored symbols). Altogether, the results show the importance of sample dilution for quantitative analysis, highlighting that dilutions between 1:50 and 1:100 were suitable for differentiating negative and positive samples without background increase and false positives.

#### Dilution of the detection antibodies

The dilution of the detection antibody must also be adjusted to avoid high backgrounds in the assay. Here we evaluated the best conditions for detection antibodies for three immunoglobulins, IgG, IgA and IgM, using S as antigen (Fig. 2G–I). Dilutions of less than 1:10,000 of anti-IgG or anti-IgA resulted in high backgrounds, clearly observed for the pre-pandemic samples (Fig. 2G,H, respectively). On the other hand, dilutions above 1:20,000 impaired the detection IgA positive samples. IgM reactivity remained detected throughout the dilution range tested (Fig. 2I). Based on the results, we chose a 1:10,000 dilution for all three detection antibodies.

#### Incubation periods for each assay step

**Figure 4:**
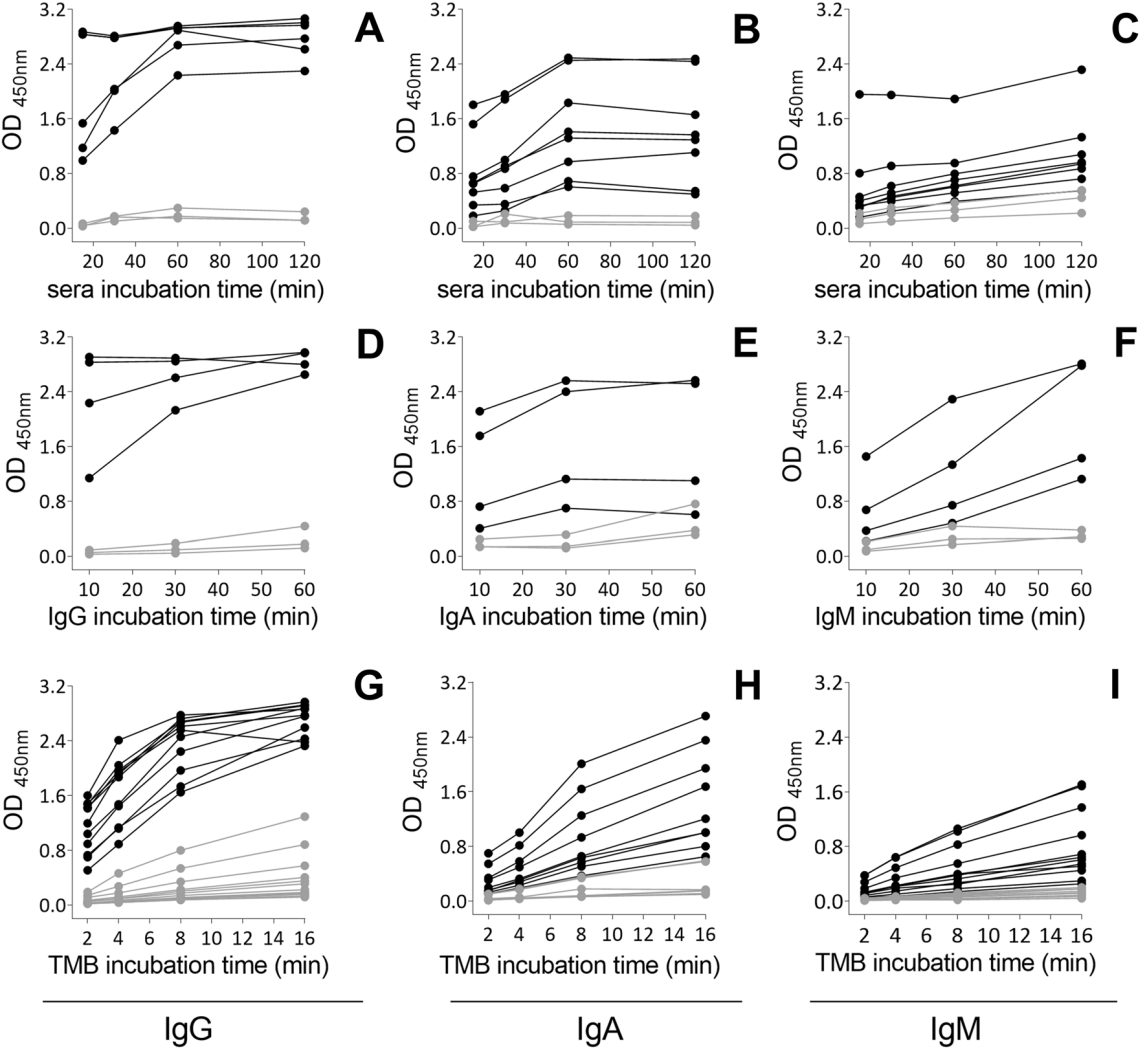
Setting the incubation time for each step of the protocol. ELISA plates were coated with 4 µg/ml S and the reactivity to sera in PCR + (black symbols) and pre-pandemic sera (grey symbols) was followed at different periods of sera incubation (**A**–**C**), detection antibody incubation (**D**–**F**) and TMB incubation (**G**–**I**) to IgG, IgA and IgM, respectively. The plates were incubated with the antigens overnight, at 4 °C, blocked for 1 h with 3% BSA in PBS-T and incubated with the sera at a 1:50 dilution. Reactivity was quantified spectrophotometrically after incubation with each detection antibody by the addition of the chromogenic substrate.

To optimize the time spent in the complete assay, we set the duration for each step of the protocol: incubation with the serum sample (Fig. 4A–C), detection antibody (Fig. 4D–F), and chromogenic substrate (Fig. 4G–I). Sera reactivity reached a plateau from 1 h of incubation under all conditions analyzed. However, differences amongst IgG positive samples were detected between 10 and 30 min of reaction. These differences were reduced or lost when the samples were incubated for 2 h, when the absorbance values exceeded the linearity limit of the assay (Fig. 4A–C). A similar profile was observed for the incubation with the detection antibody. For IgG and IgA, it was possible to differentiate positive from negative samples earlier than for IgM, for which 1 h was the most adequate incubation period for better separation between the negative and positive groups (Fig. 4D–F). Regarding the incubation time with the chromogenic substrate TMB, more pronounced differences were observed depending on the class of antibodies to be detected. For IgG, 4 min of reaction was enough to separate positive from negative samples, while for IgA and IgM, 8 min incubation was needed to obtain a proper separation of negative and positive groups (Fig. 4G–I).

We also set the conditions for plate blocking. Plate incubation for different time periods with 3% BSA solution revealed that a 10-min incubation was enough to completely reduce the background of the assay (Fig S5A). A comparison between blocking with either 3% BSA or 3% milk powder solution showed no significant difference (Fig S5B).

### Pitfalls and troubleshooting

#### Sample stability

When sera samples potentially containing infectious agents are handled, heat inactivation at 56 °C for 30 min is recommended to minimise risk of infection. Thus, to ensure assay reproducibility, it is important to evaluate temperature stability of the serum antibodies. In addition, sample collection can often be frozen and thawed for many tests. To assess serum antibodies stability to temperature variations, we performed the assay using non-inactivated and inactivated samples (Fig. S6A), as well as comparing the results obtained using fresh samples or samples subjected to 10 freeze and thaw cycles (Fig. S6B). We observed no significant differences between the reactivity of samples tested, suggesting that the antibodies present in the serum are stable under these conditions.

#### Reproducibility

The pH of the solutions used in the assay is an important factor to ensure reproducibility. To assess the effect of pH variation, we quantified the reactivity of IgG against S protein at pH 6.6, 7.4 and 8.0 (Fig. S6C). We found that at mild acidic pH (pH 6.6), 11 of 12 positive sample tested negative, with a drastic reduction in their absorbance. At weak basic pH (pH 8.0), only 1 of 12 positive sample tested negative, although the remaining 11 showed an important reduction in their absorbance. Therefore, it is crucial to carefully adjust the antigen dilution buffer to the optimal pH for the assays (pH 7.4).

The use of non-sterile buffer solutions is one of the main causes of background increase due to proteolysis of blocking solution or antigens. Therefore, the utilization of plates coated for more than 24 h should be avoided. In addition, it is recommended that all reagents are at room temperature before starting the test. Non-uniform spread of the antigen on the well during the plate coating may also contribute to large variation between replicate samples. This can be avoided by gently tapping the plate with the fingers to ensure that the entire well is covered with the coating solution. Complete removal of the blocking solution (for instance tapping the plate on a paper stack) is also important to avoid non-reproducible results. Bubbles formed during sample dilution should be removed from the assay, as they can interfere with the binding of the sample to the antigen.

### Cut-off determination and assay performance

**Figure 5:**
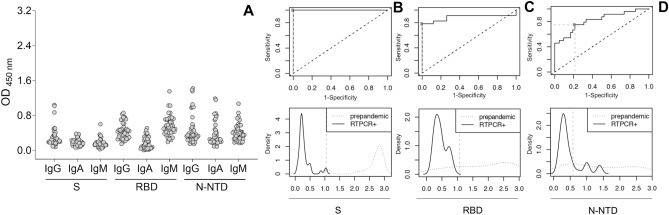
Cut-off determination in the optimized in-house ELISA. (**A**) Reactivity of IgG, IgA and IgM in 42 pre-pandemic sera to S, RBD and N-NTD. (B, C, D) Receiver operating characteristics (ROC) analysis of the optimized in-house ELISA for IgG reactivity to S (**B**), RBD (**C**) or N-NTD (**D**). Forty-two pre-pandemic sera (grey symbol) and 23 PCR + sera were diluted 1:50 in a 1% BSA solution in PBS-T and incubated for 2 h in plates previously coated with 50 μl solution of antigens at 4 μg/ml, overnight, at 4 °C, and blocked for 1 h with 3% BSA in PBS-T. For each antigen, IgG, IgA or IgM reactivity was quantified spectrophotometrically after incubation with the respective detection antibody for 1 h followed by the addition of the chromogenic substrate. AUC represents the area under the curve. In the top panels, the dotted diagonal lines represent the theoretical performance of a test with no discriminatory ability, corresponding to an AUC of 0.5. Horizontal and vertical dotted lines correspond to the sensitivity and 1-specificity, respectively, which indicates the optimal cut point value for each ELISA test, as defined by the Youden index (J) (crossed out square). In the distribution graphs (bottom panels), solid and dotted lines correspond, respectively, to PCR-positive and pre-pandemic serum samples, the vertical dotted lines indicate the optimal cut-off for each ELISA test determined by ROC analysis calculated by Youden method (Table 2).

Determination of the cut-off values is an important step to be taken when the assay is used for diagnosis purposes. Here, we first determined the sensitivity (true positive rate) and specificity (true negative rate) of the assays using as the negative group 42 sera samples collected before 2019 (pre-pandemic sera) and as the positive group, 23 sera collected from individuals who tested positive for SARS-CoV-2 infection between June 2020 and April 2021 (PCR + sera). Two different approaches were used to establish the cut-off value (Figs. 5, S7). In the first approach, it was used the three standard deviations from the mean, which is a common cut-off in practice for identifying outliers in a Gaussian or Gaussian-like distribution of ELISA results based on OD ranges^17^. In this heuristic approach, the cut-off was calculated as the mean + 3 SD of the absorbance values of the 42 sera pre-pandemic samples. Thus, PCR + samples with an OD higher than the mean of the negative controls + 3 SD were considered true positive and inversely, pre-pandemic samples with an OD higher than the mean of the negative controls + 3 SD were considered true false positive. Using this method, we identified 2 false positive samples for IgG reactivity to S and for IgA reactivity to N-NTD, corresponding to a specificity of 95.2% for the reactivity of both immunoglobulins. For IgM reactivity to the 3 antigens and for IgA reactivity to RBD, we identified 1 false positive, corresponding to a specificity of 97.6%. For IgA reactivity to S and IgG reactivity to N and RBD, no false positives were found, corresponding to a specificity of 100%.

To determine the assay accuracy, ROC analysis was performed using OD data of all ELISA assays (Fig. 5, Table 2, and suppl. Fig S7). Figure 5B–D shows the ROC analysis of the optimized assay for IgG reactivity to S, RBD, or N-NTD. The area under the curve (AUC) graphs were generated to graphically represent the performance of the assays, and the distribution graphs represent the dispersion of the PCR-positive and pre-pandemic serum samples relatively to the optimal cut-off for each ELISA. AUC values were high for all three assays, with highest value for the S-IgG ELISA (range 1–1). The Youden method that integrates both sensitivity and specificity of the test allows selecting the best cut-off values, which were estimated in a range of 0.583 to 1.073 units of absorbance (Table 2). These calculated cut-off values determined the classification of the samples (positive and negative predicted values and likelihood ratios) for subsequent analyses (Table 2). While the cut-off values calculated based on two different approaches were very similar for IgG reactivity to S and RBD, they were very different regarding N-NTD (0.53 and 1.51 units of absorbance for ROC and mean + 3 SD analyses, respectively). This apparent discrepancy is mainly due to the high dispersion of the pre-pandemic results for IgG reactivity to N-NTD (two small peaks in the distribution plot in Fig. 5D), which very likely results from false positive samples (non-specific reactivity and/or cross-reactivity to N-NTD). In addition, the low sensitivity results obtained for N-NTD, especially in the approach using the mean + 3 SD, may be explained by the absence of antibodies against this protein in the PCR + samples used in the assay. Similar results of high sensitivity and specificity were obtained for IgA reactivity to S and RBD, while poor specificity and sensitivity was observed against N-NTD (Fig. S7). Finally, IgM reactivity to S was found to be very sensitive and specific, in contrast to reactivity to RBD, which was neither specific nor sensitive (Fig. S7).

**Table 2:** Performance of the in-house ELISA using different methods of cut-off calculation.

Performance measures	In house ELISA assays		
	IgG S	IgG RBD	IgG N
**Performance of the in-house ELISA using the average OD + 3 SD of the pre-pandemic samples**			
Cut-off	0.940	1.033	1.51
Sensitivity (95% CI)	1 (0.851-NaN)	0.791 (0.578–0.928)	0.458 (0.255–0.671)
Specificity (95% CI)	0.952 (0.838–0.942)	1 (0.915-NaN)	1 (0.915-NaN)
Positive Predictive Value	0.997 (0.990–0.999)	Inf (Inf-NaN)	Inf (Inf-NaN)
Negative Predictive Value	1 (0.999-NaN)	0.201 (0.103–0.355)	0.086 (0.063–0.123)
Positive Likelihood Ratio	0.210 (0.054–0.812)	0 (0-NaN)	0 (0-NaN)
Negative Likelihood Ratio	0 (0-NaN)	0.211 (0.103–0.456)	0.541 (0.373–0.781)
**Performance of the in-house ELISA using the ROC analysis**			
AUC (95% CI)	1 (1–1)	0.885 (0.765–1)	0.818 (0.707–0.930)
AUC standard error	0	0.060	0.056
Optimal cut-off (Youden)	1.042	1.073	0.583
Sensitivity (95% CI)	1 (0.858-NaN)	0.783 (0.563–0.925)	0.750 (0.533–0.902)
Specificity (95% CI)	1 (0.916-NaN)	1 (0.916-NaN)	0.786 (0.632–0.897)
Positive Predictive Value	1 (0.862-NaN)	1 (0.824–1)	0.667 (0.484–0.860)
Negative Predictive Value	1 (0.913-NaN)	0.894 (0.750-NaN)	0.846 (0.677–0.929)
Positive Likelihood Ratio	Inf (Inf-NaN)	Inf (Inf-NaN )	3.50 (1.876–6.529)
Negative Likelihood Ratio	0 (0-NaN)	0.217 (0.100–0.472)	0.318 (0.156–0.648)

*CI* confidence interval;* NaN* not a number;* Inf* infinity;* AUC* area under curve.

### Validation of the in-house ELISA assays

**Figure 6:**
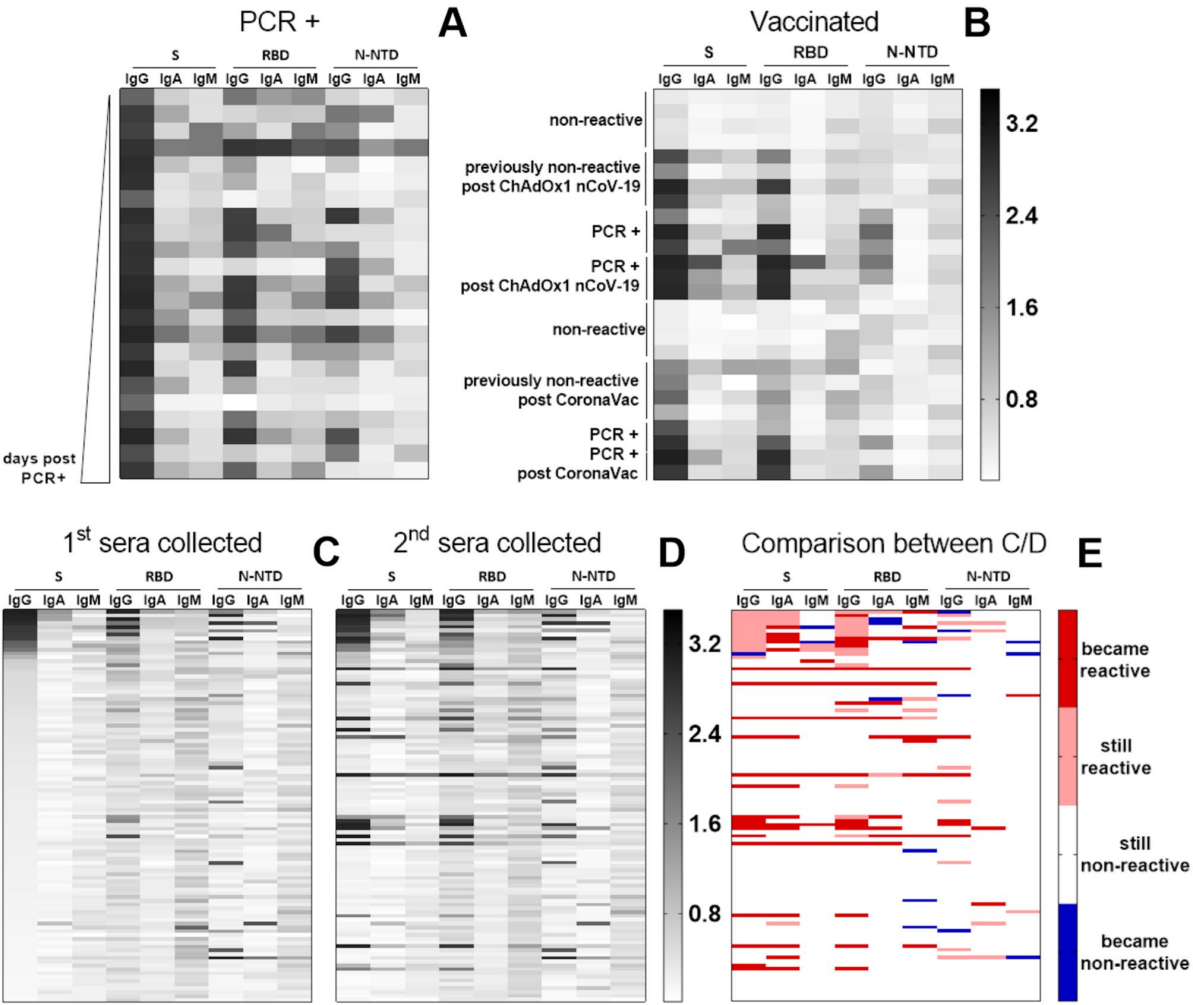
In-house ELISA application in different groups of samples. Reactivity of sera IgG, IgA and IgM against S, RBD and N-NTD. (**A**) Sera from individuals who tested positive for SARS-CoV-2 infection (PCR + samples) shown in ascending order of days post confirmed PCR; (**B**) Vaccinated individuals; (**C**) Fist sera collected in November 2020 from individuals without diagnosis for SARS-CoV-2 infection shown in ascending order of S IgG OD value and (**D**) second sera collected in April 2021; (**E**) Comparison between the results of samples analysis shown in (**C**) and (**D**). Sera were diluted 1:50 in a 1% BSA solution in PBS-T and incubated for 2 h in plates previously coated with 50 μl solution of antigens at 4 μg/ml, overnight, at 4 °C, and blocked for 1 h with 3% BSA in PBS-T. For each antigen, IgG, IgA or IgM reactivity was quantified spectrophotometrically after incubation with the respective detection antibody for 1 h followed by the addition of the chromogenic substrate.

In order to validate our in-house ELISA, we applied the complete assay to 3 different groups of samples: (i) sera collected from 23 individuals who tested positive in a PCR test for diagnosis of SARS-CoV-2 infection (*PCR* + *samples*); (ii) 206 sera samples collected from 103 individuals (2 samples from each individual, collected in October–November 2020 and February–April 2021) who were not diagnosed for SARS-CoV-2 infection and before receiving any type of vaccine for SARS-CoV-2 (*Non-diagnosed individuals*); and (iii) 26 sera samples collected from 13 individuals vaccinated against SARS-CoV-2 (*Vaccinated individuals*), being 2 samples from each individual, the first before vaccination and the second 20 days after the first dose of ChAdOx-1 nCoV-19 (7 individuals) or CoronaVac (6 individuals) vaccines (Table 1). Among these 13 individuals, 8 were not reactive for SARS-CoV-2 antibodies and 5 tested positive in a PCR test for diagnosis of SARS-CoV-2 infection before vaccination. All results obtained were grouped as a heatmap graph (Fig. 6).

#### *PCR* + *samples*

Applying the test to the PCR + samples, we found 100% IgG reactivity to S, 78% to RBD and 52% to N-NTD (Fig. 6A). The detection of IgG against the 3 antigens was observed in 47% of the samples. The 4 sera that were negative for IgG against RBD had the lowest IgG titer against S, highlighting the importance of RBD epitopes to the serological reactivity to S. IgA and IgM reactivity to S were found in 80 and 60% of the samples, respectively. Among the samples reactive to RBD, 72% were positive for IgA and 44% for IgM, while for N-NTD, 58% of the reactive samples were positive for IgA and only 8% for IgM.

#### Non-diagnosed individuals

The analysis of the serological response of the first sample collected (November 2020) of all the non-diagnosed individuals showed that 12.6% of them were positive for S IgG, 11.6% for RBD and 11.6% for N-NTD IgG. Only 4.8% of the samples were positive for all antigens (Fig. 6C). Demographic analysis of these individuals revealed that 39% (40 out of 103 individuals) reported some flu-like symptoms before November 2020 (Table 1). From this sub-group, 17% showed IgG reactivity to S and N-NTD and 11% to RBD (Fig. 6C). Among the other 63 asymptomatic individuals, only 8% showed IgG reactivity to S and RBD, while 14% were positive to N-NTD. In addition, it is important to note that 7 individuals (4 from the 63 asymptomatic individuals—9.3%—and 3 from the 40 symptomatic—5%) showed serologic response only to N-NTD IgG, which can be interpreted as a non-specific reactivity and/or a cross-reaction to N-NTD of other coronaviruses or even to proteins from other viruses that are endemic in Brazil^18^. Analyses of samples collected from those individuals (non-diagnosed in November 2020; Fig. 6E) until 5 months later, to April 2021, revealed an important seroconversion, with a 100% increase in S and RBD IgG incidence and a 16% increase in N-NTD IgG detection (Fig. 6D). Differently from the PCR + samples, only 30% of the positive sera for IgG against any of the 3 antigens were also positive for IgA, and only 20% for S IgM and 8% for RDB and N-NTD IgM. Very few samples showed only IgA or IgM reactivity.

#### Vaccinated individuals

As the vaccination progress, seroconversion from SARS-CoV-2 infection overlaps with the serological response to vaccines. To illustrate the potential differences that can be detected by assessing serological responses at a time when a substantial part of the population is vaccinated, we performed our in-house using serum samples collected from individuals who tested positive for SARS-CoV-2 infection or from non-reactive individuals before and after receiving the first dose of CoronaVac or ChAdOx-1 nCoV-19 vaccines (Fig. 6B). CoronaVac is an inactivated virus vaccine, so that the individual is immunized by the contact with all the viral proteins^19^. In contrast, ChAdOx-1 nCoV-19 vaccine consists of an adenoviral vector containing the full-length SARS-CoV-2 S protein^20^. As expected, all sera from vaccinated individuals were positive for S IgG, but, for these analyzed samples, IgG reactivity to RBD was more evident in PCR + individuals after immunization for both vaccines. Despite containing N protein, CoronaVac did not induce a significant N-NTD IgG response in the tested samples. ChAdOx-1 nCoV-19, but not CoronaVac, increased the S IgA titer in the subjects previously diagnosed with SARS-CoV-2 infection and induced this response in individuals who were previously not-reactive. Only 1 PCR + individual showed IgA reactivity to RDB after receiving ChAdOx-1 nCoV-19. No induction of IgM response was seen for any of the three antigens after immunization with any of the vaccines.

## Discussion

In this study we developed a serological test with high sensitivity and specificity to assess the presence of the three main immunoglobulin isotypes targeted against the main antigens routinely used in Covid-19 diagnosis. Serological tests may represent important tools not only for serosurveillance but also for monitoring vaccination coverage and the duration of immune response to the vaccine^21^, being especially interesting in a country such as Brazil, which shows the highest number of confirmed cases of Covid-19 in Latin America although it has started mass vaccination of its population against Covid-19 since January 2021. One advantage of the present test is that it can be customized in different stages according to its application, enabling the user to save time, reagents, and/or samples, as well as to use it for the analysis of different cohorts. This flexibility is useful since multiplexing the test increases its efficiency and facilitates antigen substitution (e. g. by including mutations present in circulating variants). Furthermore, the overall cost compared to those of commercial kits is considerably reduced.

Several studies have shown the importance of combining more than one antigen to increase the efficiency of serological assays^13,22^. Here, we selected the antigens considering the strong immune response to N-NTD^23^ and the high specificity of the response to S^24^ and RBD^25^. Moreover, the level of antibodies directed to RBD strongly correlates with a neutralizing response^14,26,27^, providing useful information when handling the viral particle is not possible. Furthermore, the combination of three antibody isotypes (IgG, IgA and IgM) improves the efficiency of using the test for diagnostic purposes^28^. While IgM may represent a recent infection complementing the molecular diagnosis for SARS-CoV-2^29^, a recent study showed a high incidence of IgA response in PCR + samples, even when they did not show IgM reactivity^30^.

One of the main obstacles in the development of an in-house serological test for SARS-CoV-2 is to evaluate the diagnostic performance and cut-off value of the assay, as reliable positive (PCR +) and negative (pre-pandemic) samples are required. Since the emergence of the virus is quite recent and the appearance of new variants is frequent and unpredictable, the details of the humoral immune response to its antigens are still unknown. In continuous diagnostic clinical testing, establishing and maintaining a reliable threshold is crucial to discriminate between infected (true positive) and uninfected (true negative) subjects. Particularly for in house ELISAs, the cut-off value may be defined as the average + 3 SD of the absorbance values obtained for the negative samples, a common approach used in many studies^24,31^. On the other hand, threshold value calculation using ROC curves represents the ad hoc methodology used in most diagnostic tests, allowing to assess test performance and robustness^32^. The difficulty in determining cut-off values for the SARS-CoV-2 serological tests can be clearly illustrated by the analysis of our pre-pandemic samples. We observed a high reactivity for IgG and IgM against RBD in a substantial number of samples. Conversely, a previous report showed high specificity for the serological response against RBD^25^, suggesting that negative samples’ particularities may generate different results. This discrepancy reinforces how we are still far from a complete understanding of the immune response against SARS-CoV-2. We also detected non-specific reactivity to the three antibody isotypes against N-NTD in the pre-pandemic samples. Accordingly, other studies have reported cross-reactivity between SARS-CoV-2 and other coronaviruses’ N proteins, including SARS-CoV^33^ and MERS-CoV^34^. This is expected given the high homology between the amino acid sequence of these proteins, which is 90.5% and 46.1%, respectively^33^. Additionally, cross-reactivity among SARS-CoV-2 and the alpha-coronaviruses HCoV-NL63 and HCoV-229E N proteins has also been reported. Since amino acid sequence homology in much lower in this case, it is possible that cross-reactivity may also result from protein similarities at the conformational level^35^. It should also be considered that Brazil is a country where dengue virus (DENV) is endemic and a possible cross-reactivity between DENV and SARS-CoV-2 antibodies has already been reported^36^. In addition, infection by other arboviruses, such as the Zika virus, may contribute to false positive results, as reported for the application of a commercial SARS-CoV-2 ELISA test in patients with acute Zika virus infection^18^. Therefore, further analysis of the impact of arbovirus infections on SARS-CoV-2 serological tests is needed. Interestingly, false negative results were observed in PCR-positive samples, which was particularly the case of IgM and IgG reactivity against N-NTD in our test. This undetected reactivity could be explained by late sampling of these immunoglobulin isotypes, which are detected very early after the onset of symptoms^33^.

The validation of the assay presented here was performed using 23 sera from PCR + individuals, with 100% of the samples positive for IgG against S and 78% against RBD. Interestingly, samples that did not show IgG response against RBD presented low S IgG titers. Regarding the response to N-NTD, 52% of our PCR + samples were classified as reagent for N-NTD IgG. This relatively low reactivity for N-NTD would be explained by the fact that serological response to N protein tends to occur earlier^37,38^, and our PCR + cohort is composed of samples collected in a range from 20 to 157 days after PCR confirmation. In the case of IgM response, we found a low incidence for samples positive for S and RBD, and an even rarer reactivity for N protein. Indeed, the incidence of IgM against protein N seems to be lower than for other antigens^29^, so that the analysis of this response in samples collected at the onset of the symptoms should be considered. It is also important to consider that here we analyzed samples from individuals who presented mild symptoms and were fully recovered, which may explain the lower incidence observed for some immunoglobulin isotypes, as antibody titers strongly correlates with disease severity^39–41^. Indeed, studies performed with hospitalized patients have reported up to 94% seroconversion of against N protein^42–44^.

Among the participants of the present study, 63 were asymptomatic individuals, from whom the samples of 8% were identified as positive for IgG reactivity to S and RBD. Furthermore, in the symptomatic non-diagnosed group of our cohort, 17% of the individuals who seroconverted reported only mild symptoms. This result reinforces the robustness of the test, especially in this type of cohorts, since some studies have not detected SARS-CoV-2 antibodies in asymptomatic individuals or individuals with mild and short duration symptoms^45,46^. Interestingly, for the group of individuals who were not diagnosed for SARS-CoV-2, we investigated samples collected at two time points before vaccination (November 2020 and April 2021). We found a twofold increase in the incidence of positive cases in the second sample when compared to the first sample. These data are consistent with the increased incidence of new daily cases observed in the state of Rio de Janeiro (Brazil) during this period. In November 2020, an incidence of up to 1500 new cases per day was reported, while in April 2021, 3000 new daily cases were reported^47^.

There are currently different types of vaccines in clinical use. In the current pandemic phase, seroconversion can result either from contact with the virus or from vaccinal immunization. Here we applied our assay to sera from individuals with or without a previous SARS-CoV-2 infection, who were vaccinated with CoronaVac (a purified inactivated SARS-CoV-2 vaccine)^19^, or ChAdOx1 nCoV-19 (the adenovirus-vectored vaccine encoding SARS-CoV-2 spike protein)^20^. Due to the contact with all the viral proteins in vaccination with CoronaVac, a serological response to protein N is expected and detected in other studies^19,48^, although we did not observe this result in the tested samples. On the other hand, the response against N-NTD can be useful to identify pre-vaccination infections.

Finally, despite the very satisfactory effectiveness of vaccines to reduce the worsening of symptoms and the number of deaths associated with Covid-19, it is also believed that a high percentage of immunization in the world’s population will be necessary to prevent the chain of virus transmission and avoid the appearance of new variants. As we currently face the extension of the pandemic, health agencies are recommending booster doses for the population. Heterologous immunization (the same individual receiving different immunizations at different doses) seems to represent an important tool to increase effectiveness of immunization^49^. In this context, the serological test developed here allows the identification of diverse immune responses to various vaccine antigens, enabling a more precise understanding of the different immunization regimens across more specific populations (age, sex, comorbidities and so on). It is known that antigen polymorphism of pathogens has the potential to reduce the performance of the serological diagnostic tests. The emergence of new variants, as the recent Omicron variant, accumulating a high level of mutations in both spike and RBD could impair the sensibility of serodiagnosis tests of SARS-CoV-2. Omicron RBD, which contains 15 mutations in the RBD, shows a significantly weaker binding to antibodies of human serum from SARS-CoV-2 vaccinated and Covid-19 patients (alpha variant) in comparison to the RBD of the original Wuhan strain^50^. Thus, the use of a diagnostic test with a multiplex strategy using different antigens with different selective pressure has a clear advantage over tests using only RBD as antigen. Therefore, we believe that our multiplex ELISA test represents a valuable tool for defining more effective public health policies to fight this deadly contagious disease, thus contributing to the definition of vaccine strategies to be used in the future.

## Limitations

In this article we present a customizable ELISA-based test to detect IgG, IgA and IgM against three SARS-CoV-2 proteins: trimeric S, RBD and N-NTD. This assay allows the user to analyze different cohorts, saving time, reagents, and samples. One of the main limitations of this assay is the need of a cohort of pre-pandemic samples to establish reliable cut-offs. Although the assay allows to easily distinguish between positive and negative samples, even when the positive samples have lower antibodies’ titers, sera titration experiments may be required to access differences between samples with higher antibodies’ concentrations. The correlation between the reactivity against N protein and the actual contact with SARS-CoV-2 (or its components, in the case of whole virus vaccines) is still controversial and consists in a major limitation. In some cases, individuals previously infected with SARS-CoV-2 do not show a detectable response against N protein. Therefore, it is possible that some samples classified as N protein-reactive are resultant of serum cross-reactivity to proteins from other coronaviruses or even from viruses of other families.

## Methods

### Antigens’ expression and purification

#### S protein

The trimeric S protein produced in HEK293-3F6 cells, was kindly provided by Dr. Leda Castilho, COPPE, UFRJ)^24^.

#### Receptor-binding domain (RBD)

RBD expression was adapted from a previously described protocol^51^. HEK293T cells were transfected with pcDNA3.1( +) vector (GenScript, New Jersey, USA) containing residues 319 to 541 of SARS-CoV-2 S protein using PEI MAX 40 K (Polysciences, Warrington, USA) in a 3:1 ratio of PEI to DNA. The day before transfection, cells were seeded on 100 mm dishes at a density of 4.0 × 10^6^ cells with viability greater than 95%. The cells were maintained in Opti-MEM medium (Gibco, Massachusetts, USA) supplemented with 5% fetal bovine serum (FBS; Invitrogen, Massachusetts, USA) and 1 mM sodium pyruvate (Gibco), in a CO_2_ humid incubation chamber, at 37 °C. Next day, PEI:DNA complexes were prepared with 16 µg of plasmid DNA for each cell dish in serum-free Opti-MEM, incubated for 20 min at room temperature (RT) and added to the cell culture. Cells were maintained in a CO_2_ humid incubation chamber, at 37 °C, overnight. Then, the culture medium was removed, the cells were washed gently with phosphate-buffered saline (PBS) to remove residual FBS and the medium was replaced with a serum-free Opti-MEM supplemented with 4 mM L-glutamine (Sigma-Aldrich, Missouri, USA) and 1 mM sodium pyruvate (Gibco). The supernatant containing the expressed proteins was collected 72 h after transfection, centrifuged for 20 min at 4,000 g, 4 °C and filtered through a 0.22 μm filter to remove cells and debris. Phenylmethylsulfonyl fluoride protease inhibitor (PMSF; Sigma-Aldrich) has been added to prevent protein degradation. The supernatant was purified on a HisTrap FF column (GE Healthcare Life Sciences, New Jersey, USA) by immobilized metal affinity chromatography (IMAC) using IMAC equilibration buffer (50 mM Tris–HCl (pH 8), 500 mM NaCl, 20 mM imidazole, 10% glycerol) and IMAC elution buffer (50 mM Tris–HCl (pH 8), 500 mM NaCl, 500 mM imidazole, 10% glycerol). The protein was eluted with a linear 20–500 mM imidazole gradient. For buffer exchange and protein concentration, the eluate was placed in a Amicon Ultra-4 3 K Centrifugal Filter Device (Merck Millipore, Massachusetts, USA). The device was loaded with a RBD:PBS 1:3 v/v ratio and centrifuged at 4,500 g at 4 °C until only 500 µl remained in the unit. This step was repeated twice. Sample purity was confirmed by 15% SDS–PAGE gels. The expression, purification and protein analysis can be found in supplementary material.

#### N protein N-terminal domain (NTD)

*Escherichia coli* BL21 (DE3) was transformed with a pET28a His-tagged construct (GenScript New Jersey, USA) encoding for the amino acid residues 44 to 180 of the N-terminal RNA-binding globular domain of the Nucleocapsid protein, N (N-NTD)^52^. Bacteria were grown in LB medium with 50 μg/ml kanamycin. Protein expression was induced with 0.2 mM IPTG when optical density (OD) reached 0.6–0.8 at 600 nm. After 16 h of induction at 16 °C, cells were centrifuged, pelleted resuspended and disrupted by ultrasonication in lysis buffer: 50 mM Tris–HCl (pH 8), 500 mM NaCl, 20 mM imidazole, 10% glycerol, and 43 mg/L of protease inhibitor cocktail (P8465—Sigma-Aldrich, Burlington, MA, USA). The lysate was centrifuged at 12,000 g for 60 min at 4 °C. The supernatant was filtered and applied to an HisTrap FF affinity column (GE Healthcare Life Sciences, New Jersey, USA). N-NTD recombinant protein was purified by IMAC as described previously for RBD. For the preparation of the sample used in this study, His-tag was removed by an overnight cleavage with TEV protease (TEV:protein 1:10 molar ratio) during dialysis against 50 mM Tris–HCl buffer, pH 8, containing 500 mM NaCl and 1 mM DTT, followed a reverse IMAC at a flow rate of 1 ml/min. However, to the best of our knowledge, we are unaware of evidence indicating that the His-tag tail interfere with the assay. At the end of the purification, 500 µM PMSF, 3 mM sodium azide, and 3 mM EDTA were added to pooled fractions containing the protein. The purity of the samples was confirmed by 18% SDS–PAGE gels.

### Sera samples

A total of 240 blood samples were collected from 126 individuals in the Laboratory of Clinical Analysis of the School of Pharmacy at Federal University of Rio de Janeiro (UFRJ). All participants completed a written informed consent and answered a survey providing data on demographics, medical history, symptoms, and history of travel abroad before enrolled in the study, which was approved by the local ethics committee (CEP HUCFF/UFRJ approval #35,303,120.5.0000.5257). The cohort was divided into two groups: (i) samples collected from 23 individuals who tested positive in a PCR assay for SARS-CoV-2, between April 2020 and January 2021, before enrolling in the study (PCR +); and (ii) samples collected form 103 individuals without confirmed diagnosis for SARS-CoV-2 infection, from whom samples were collected twice, firstly between October and November 2020 and then between February and April 2021. From these two groups, 7 individuals from the PCR + group and 11 individuals from the undiagnosed group were selected for blood collection 20 days after they received the first dose of ChAdOx1 nCoV-19 or CoronaVac vaccines. All the sera samples were aliquoted, heat inactivated at 56 °C for 30 min and stored in − 80 °C until further analysis. Additionally, pre-pandemic samples collected from 42 individuals before 2019 used as negative controls.

### Enzyme-linked immunosorbent assay (ELISA)

The assay presented here was adapted from a previously reported protocol^16^. Reactivity of types of sera antibodies—IgG, IgA, and IgM—against three SARS-CoV-2 antigens—trimeric Spike (S), RBD and N-NTD was analyzed. Briefly, each 96-well plate (Corning™ Costar™, Life Science, New York, USA) was coated with 50 μl per well of one of the antigens and incubated at 4 °C overnight. After blocking, 80 μl serum samples (heat-inactivated at 56 °C for 30 min) were added per well. The presence of reactive IgG, IgA or IgM in sera samples was detected using mouse anti-human IgG (Fc specific), goat anti-human IgA (α-chain specific), or goat anti-human IgM (μ-chain specific), respectively, conjugated with HRP (Sigma-Aldrich, Missouri, USA). Sera reactivity was developed using the chromogenic reagent 3,3′,5,5′-Tetramethylbenzidine dihydrochloride (TMB substrate solution, Thermo Fisher, Massachusetts, USA). Absorbance values at 450 nm were determined on a VICTOR Multilabel plate reader (PerkinElmer).

### Cut-off calculations

The cut-off values for each antigen and each detection antibody were first determined using the average OD plus 3 standard deviations (SD) of the 42 pre-pandemic samples. The evaluation of the performance of the tests was assessed by calculating sensitivity, specificity, positive and negative predicted values with their 95% confidence intervals using Prism v8 (GraphPad, USA). Alternatively, the receiver-operating characteristic (ROC) curve was generated for each test to define the optimal threshold cutoff value that distinguishes positive from negative samples. The area under the curve (AUC), sensitivity and specificity for all thresholds were determined using the online calculator easyROC^53^. Then, the optimal cutoff point was selected based on the point with the highest Youden index J (J = sensitivity + specificity − 1). This is the point on the curve where the distance to the diagonal line (line of equality) is maximum.

### Statistical analysis

Statistical analyses were performed using Prism v8 (GraphPad, USA) and easyROC online web-tool for ROC curve analysis (ver. 1.3.1)^53^.

### Ethics approval

This study was performed according to the principles of the Declaration of Helsinki under approval by the local ethics committee CONEP/CEP HUCFF/UFRJ #35303120.5.0000.5257.

### Consent to participate

Informed consent was obtained from each participant included in this study.

## Supplementary Information

Supplementary Information.

## Data Availability

The original datasets and analyses of this study are available in the figshare repository 10.6084/m9.figshare.19203893.
