# Supplementary material for: On the caveats of a multiplex test for SARS-CoV-2 to detect seroconversion after infection or vaccination
Source: Sci Rep. 2022 Jun 20;12:10366. doi: 10.1038/s41598-022-14294-8 (PMC9208546; doi:10.1038/s41598-022-14294-8)
Supplement: Supplementary file 1 — Supplementary Information. [file 41598_2022_14294_MOESM1_ESM.pdf]

On the caveats of a multiplex test for SARS-CoV-2 to detect seroconversion after infection or vaccination

Supplementary information

- Antigens produced for the assay

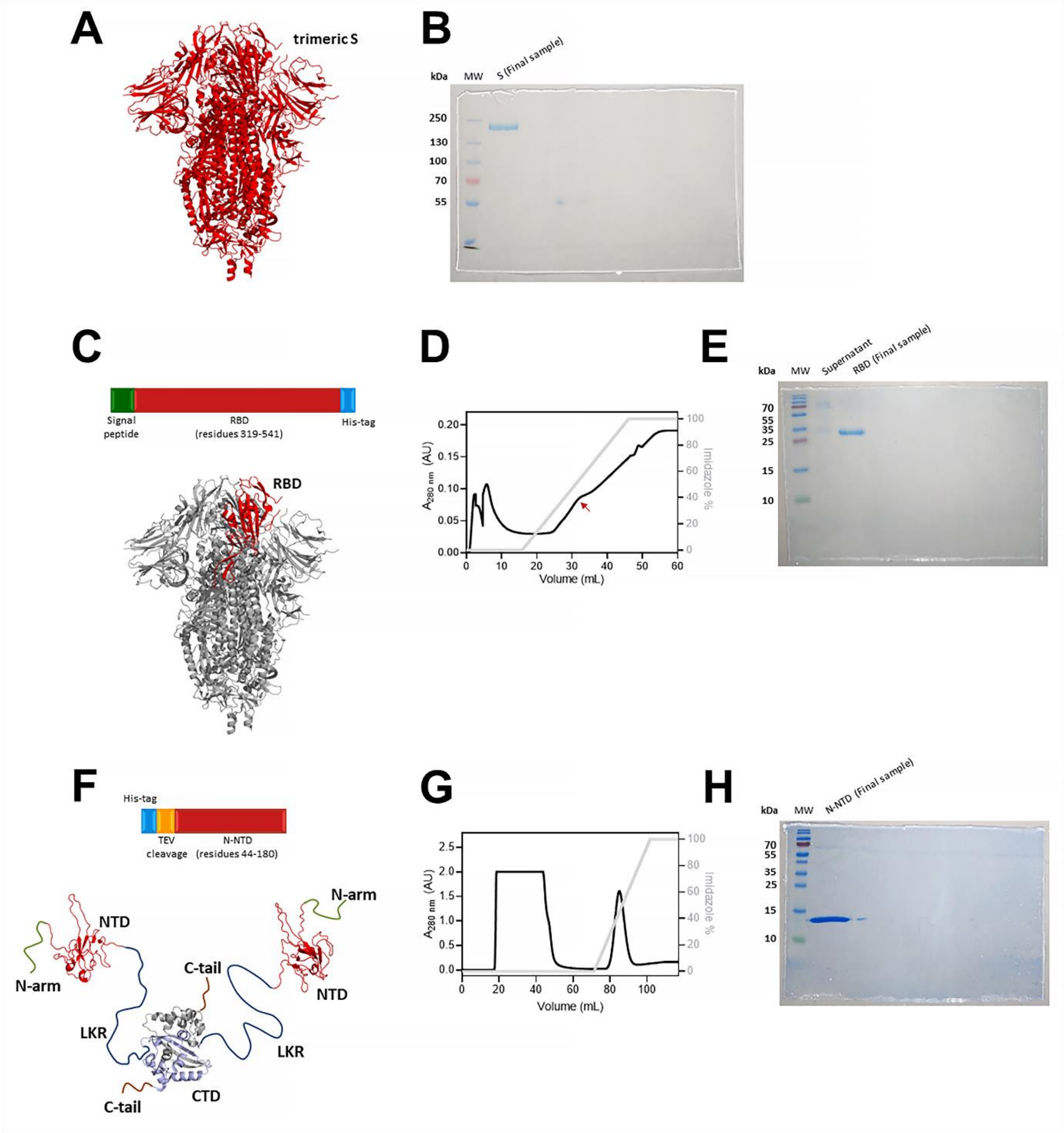

Figure S1: Recombinant proteins produced for the assay. Three antigens were produced for the assay: S protein (A, B), the receptor binding domain (RBD; C-E), and the N-terminal domain of N protein (N-NTD; F-H). Trimeric S (PDB ID 6VXX; A) produced in HEK293 cells adapted to grown in suspension and purified by affinity chromatography was kindly provided by PhD Leda Castilho (COPPE, UFRJ). The high purity of S preparation is confirmed by SDS-PAGE (B). Recombinant RBD construct comprising residues 319-541 of SARS-CoV-2 S protein

(red) was cloned with a hexa-histidine tag (His-tag) (blue) and signal peptide sequence (green) (C). In the lower panel of (C), the ribbon representation of the trimeric SARS-CoV-2 S (gray) with RBD highlighted (red) was generated with PyMOL using PDB ID 6VXX. RBD was purified using a HisTrap FF column, eluted with an imidazole gradient, as shown in the representative elution chromatogram (D), in which the peak indicated by the red arrow in the imidazole gradient (gray line) corresponds to the pure protein. The high purity of the preparation is confirmed by SDS-PAGE (E). Recombinant N-NTD construct comprising residues 44-180 of N protein (red) was cloned with a hexa-histidine tag (His-tag) (blue) and a TEV cleavage sequence (yellow) (F). In the lower panel of (F), the ribbon representation of N protein dimer was prepared with PyMOL using PDB IDs 6YI3 and 6WZO. N-NTD was purified using a HisTrap FF column, eluted with an imidazole gradient, as shown in the representative elution chromatogram, in which the peak in the imidazole gradient (gray line) corresponds to the pure protein with His-tag (G). For the preparation of the sample used in this study, His-tag was removed by cleavage with TEV protease, although there is no evidence indicating that the His-tag tail interferes with the assay. Cleaved protein was obtained in the flow-through of a reverse IMAC, and the high purity preparation is confirmed by SDS-PAGE (H).

- Complementary results to Figure 2: Reactivity of sera IgA and IgM as a function of antigen coating density

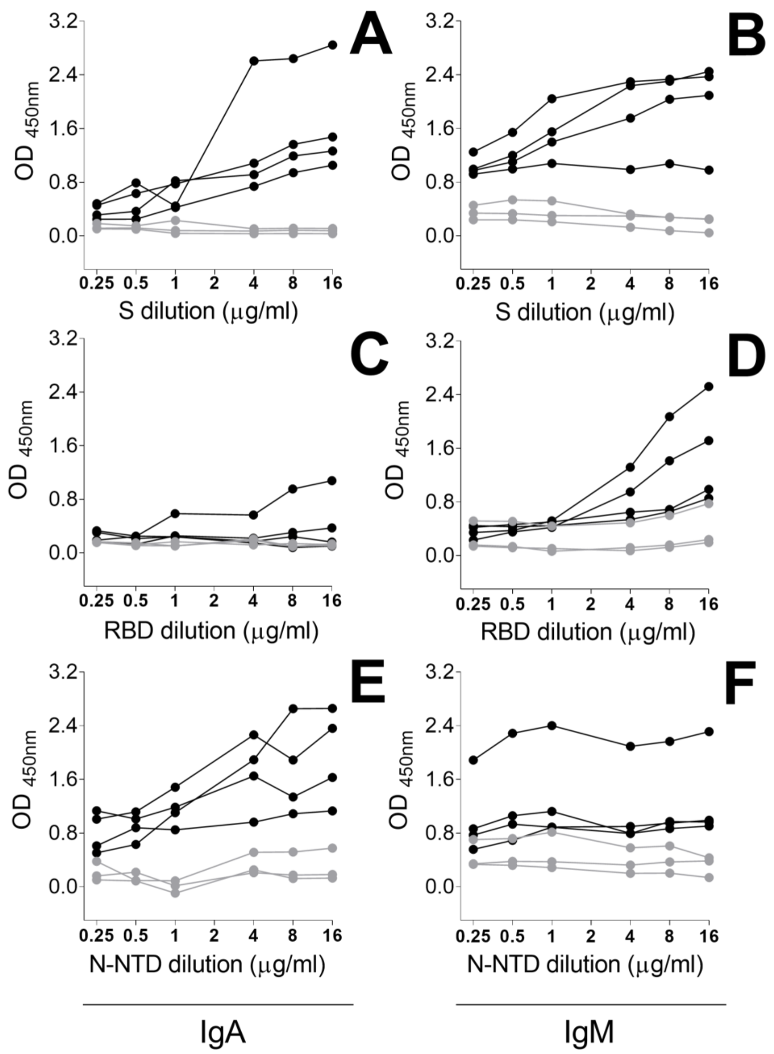

Figure S2: Sera reactivity to SARS-CoV-2 antigens as a function of the plate coating density. S protein (A,B), RBD (C,D) or N-NTD (E,F) were serially diluted in PBS to coat ELISA plates. The plates were incubated with the

antigens overnight, at 4°C, blocked for 1 h with 3% BSA in PBS-T and incubated for 2 h with 3 pre-pandemic sera (grey symbols) or 4 PCR+ sera (black symbols) at a 1:50 dilution. For each antigen, IgA (A,C,E) or IgM (B,D,F) reactivity was quantified spectrophotometrically after incubation with the respective detection antibody for 1 h followed by the addition of the chromogenic substrate.

- Contribution of RBD to sera reactivity to S

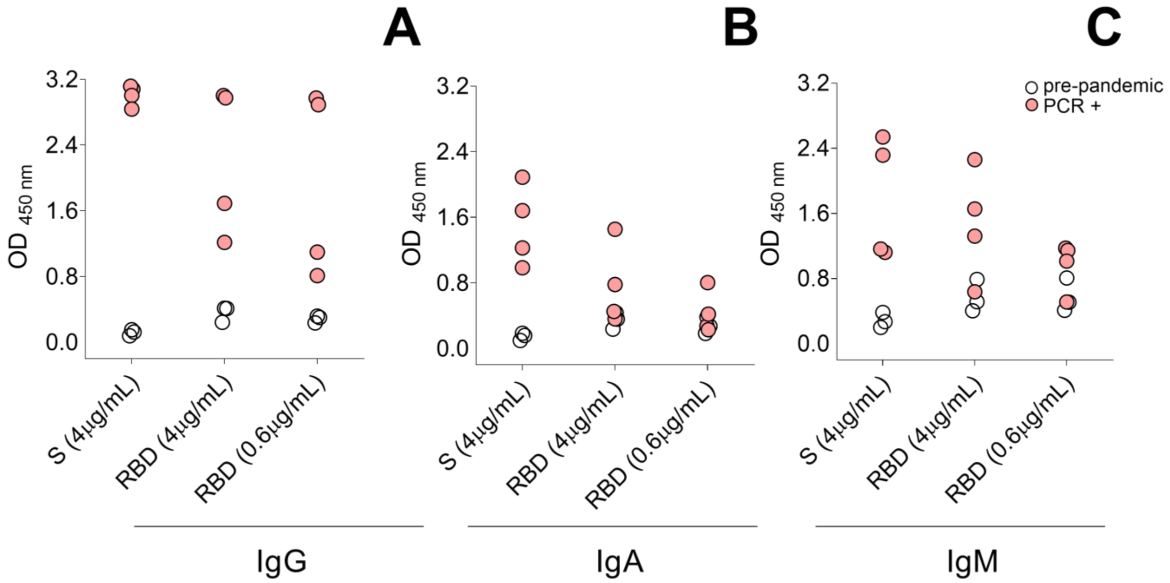

Figure S3: Contribution of RBD to sera reactivity to S protein. The plates were coated with 4 µg/ml S protein, 4 µg/ml RBD, or 0.6 µg/ml RBD. After incubation overnight, at 4°C, the plates were blocked for 1 h with 3% BSA in PBS-T and incubated for 2 h with pre-pandemic sera (grey-open circles) or 4 PCR+ sera (pink-filled circles) at a 1:50 dilution. For each antigen, IgG (A), IgA (B) or IgM (C) (as indicated) reactivity was quantified spectrophotometrically after incubation with the respective detection antibody for 1 h followed by the addition of the chromogenic substrate.

- Complementary results to Figure 2: Reactivity of sera IgA and IgM as a function of sera dilution

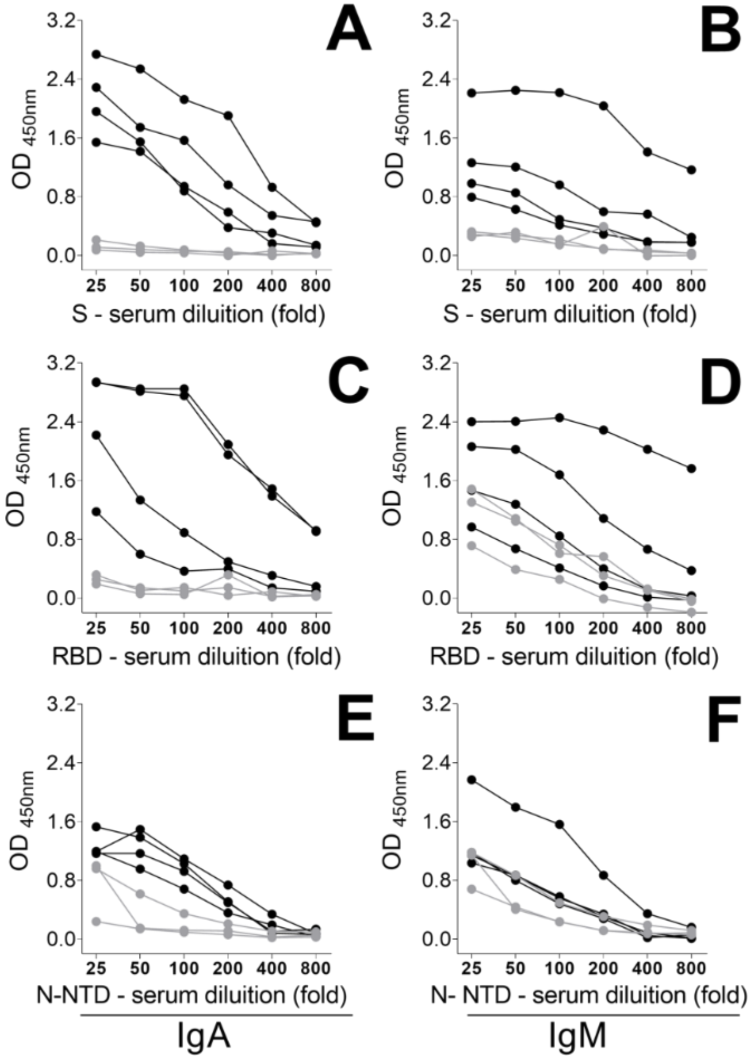

Figure S4: Sera reactivity to SARS-CoV-2 antigens as a function of sera dilution. Three pre-pandemic sera (grey symbols) or 4 PCR+ sera (black symbols) were serially diluted in a 1% BSA solution in PBS-T and incubated for 2 h in plates previously coated with 50 µl solution of S protein (A,B), RBD (C,D) or N-NTD (E,F) at 4 µg/ml, overnight, at 4°C, and blocked for 1 h with 3% BSA in PBS-T. For each antigen, IgA (A,C,E) or IgM (B,D,F) reactivity was quantified spectrophotometrically after incubation with the respective detection antibody for 1 h followed by the addition of the chromogenic substrate.

- Setting the conditions for plate blocking

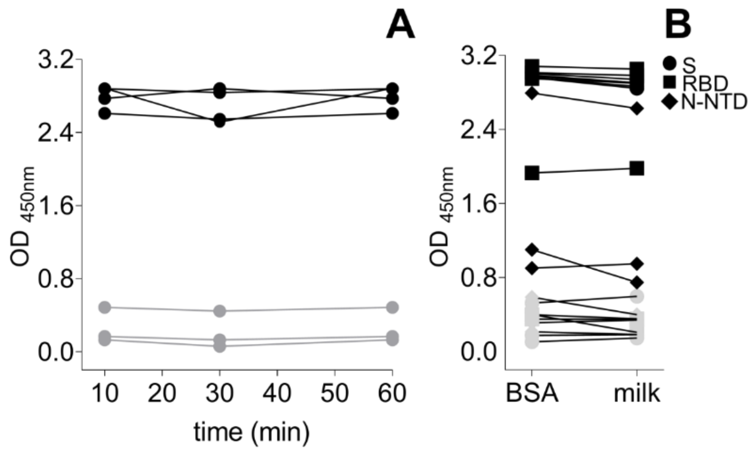

Figure S5: Setting of the blocking conditions. S, RBD or N-NTD, as indicated, were used as antigens to coat ELISA plates at 4 µg/ml, overnight, at 4°C. (A) Plates were blocked for different periods with 3% BSA in PBS-T. (B) Plates were blocked for 1h with 3% BSA or 3% milk powder in PBS-T, as indicated, and sera IgG reactivity was quantified as described in (A). Pre-pandemic (grey symbols) or PCR+ (black symbols) samples at 1:50 dilution were incubated for 2 h and IgG reactivity was quantified spectrophotometrically after incubation with the respective detection antibody for 1 h followed by the addition of the chromogenic substrate.

- Effects of temperature on sample stability and pH variations on assay sensitivity

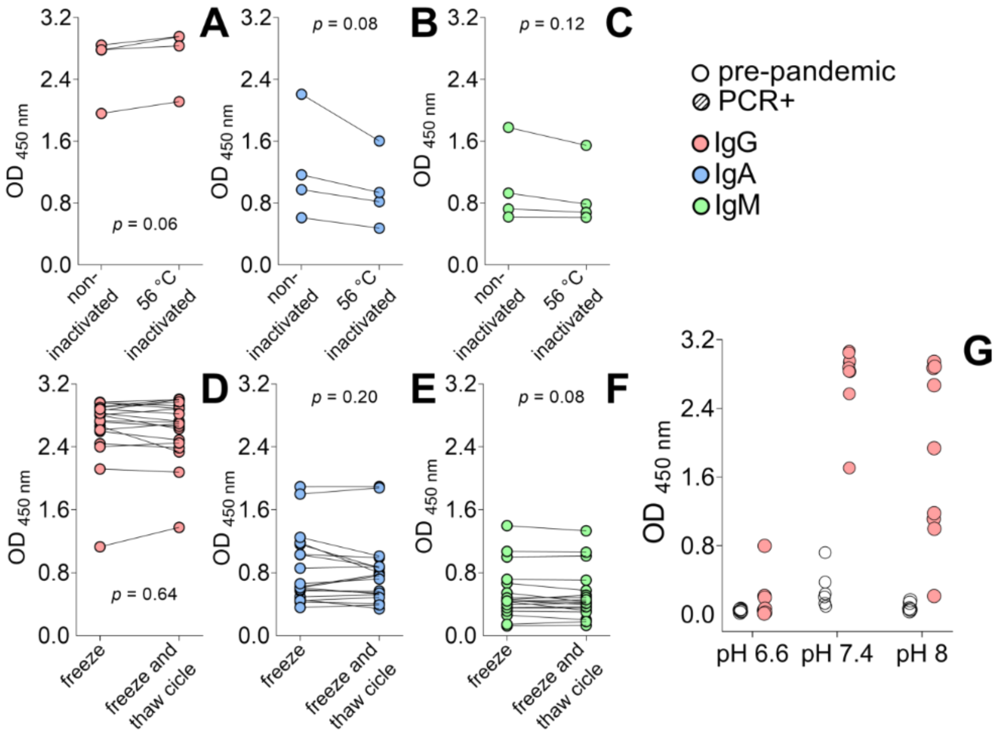

Figure S6: Conditions affecting sera reactivity to SARS-CoV-2 antigens. ELISA plates were coated with S at 4 µg/ml, overnight, at 4°C. Sera samples from PCR+ individuals were assayed with or without previously heat inactivation

at 56°C for 30 minutes (A-C), or after subjected or not to 10 freeze and thaw cycles (D-F). IgG (A, D - pink symbols), IgA (B, E -blue symbols) or IgM (C, F - green symbols) reactivity was quantified spectrophotometrically after incubation with the respective detection antibody for 1 h followed by the addition of the chromogenic substrate. (G) Three pre-pandemic sera (black-open circles) or 4 PCR+ sera (pink-filled circles) were assayed using all buffers at pH 6.6, pH 7.4 or pH 8.0. IgG reactivity was quantified spectrophotometrically after incubation with the respective detection antibody for 1 h followed by the addition of the chromogenic substrate.

- Complementary results to Figure 5: ROC analysis

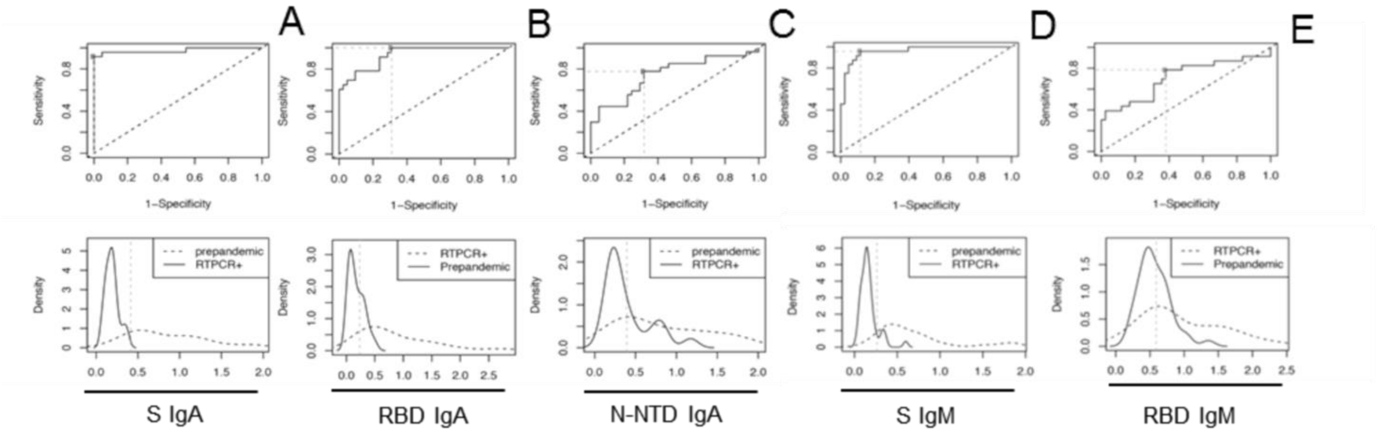

Figure S7: Receiver operating characteristics (ROC) analysis of the optimized in-house ELISA for IgA S (A), RBD (B), NTD-N (N), IgM S (D) and IgM RBD (E). Forty-two pre-pandemic sera (grey symbol) and 23 PCR+ sera were diluted 1:50 in a 1% BSA solution in PBS-T and incubated for 2 h in plates previously coated with 50 µl solution of antigens at 4 µg/ml, overnight, at 4°C, and blocked for 1 h with 3% BSA in PBS-T. For each antigen, IgG, IgA or IgM reactivity was quantified spectrophotometrically after incubation with the respective detection antibody for 1 h followed by the addition of the chromogenic substrate. AUC represents area under the curve. In the graphs above, the dotted diagonal lines represent the theoretical performance of a test with no discriminatory ability, corresponding to an AUC of 0.5. Horizontal and vertical dotted lines correspond to the sensitivity and 1-specificity, respectively, which indicates the optimal cut point value for each ELISA test, as defined by the Youden index (J) (crossed out square). In the distribution graphs below, solid and dotted lines correspond, respectively, to PCR-positive and pre-pandemic serum samples, the vertical dotted lines indicate the optimal cut-off for each ELISA test determined by ROC analysis calculated by Youden method (Table 2).
